# Relación entre espacios primate y la forma de arcos dentarios en niños de 3 a 6 años. Un estudio transversal

**DOI:** 10.21142/2523-2754-1203-2024-204

**Published:** 2024-09-17

**Authors:** Cindy Minelly Arévalo Pinedo, Guido Alberto Perona-Miguel de Priego

**Affiliations:** 1 Postgrado de Odontopediatría, Universidad Católica Santo Toribio de Mogrovejo. USAT. Chiclayo, Perú. cimiap13@gmail.com, gperona@usat.edu.pe Universidad Católica Santo Toribio de Mogrovejo Postgrado de Odontopediatría Universidad Católica Santo Toribio de Mogrovejo. USAT Chiclayo Peru cimiap13@gmail.com gperona@usat.edu.pe

**Keywords:** espacios primate, forma de arco, dentición, arcada dental, primate spaces, arch shape, dentition, dental arch

## Abstract

El presente estudio tuvo como objetivo determinar la relación entre espacios primate y la forma de arcos dentarios en los niños de 3 a 6 años matriculados en el año escolar 2023, en la I. E. Inicial Niño Jesús de Praga, de la ciudad de Moyobamba, Perú.

**Materiales y métodos:**

Este estudio prospectivo, transversal, descriptivo y observacional estuvo conformado por 237 participantes, quienes fueron examinados aplicando una ficha clínica donde se registraron los hallazgos en función de la ausencia o presencia de espacios primate, y la forma del arco dentario fue clasificada en ovalado, cuadrado o triangular. Se realizó un análisis univariado de frecuencia y porcentaje, y bivariado mediante la prueba de chi-cuadrado, con un nivel de significancia de 0,05.

**Resultados:**

Se encontró que, en la arcada superior, el 82,3% presentó espacios primates en ambos cuadrantes; en la arcada inferior fue el 50,2%, seguido por el 45,6% que mostró ausencia de espacios primate en ambos cuadrantes. La forma de arco dentario más frecuente en ambas arcadas fue la ovalada (70,5%). El 58,6% presentó espacios primate en ambos cuadrantes y forma de arco ovalada para la arcada superior, y el 34,6% para la inferior. Se encontró una relación estadísticamente significativa entre espacios primate y forma de arco dentario en la arcada superior (p = 0,005), en los participantes de 4 años (p = 0,025) y de sexo femenino (p = 0,002).

**Conclusiones:**

La presencia de espacios primate en ambos cuadrantes fue predominante para la arcada superior e inferior, así como la forma de arco ovalado, siendo la combinación de estas características la más frecuente, con lo que se espera que el desarrollo de su oclusión sea armonioso.

## Abstract (en)

The objective of this study was to determine the relationship between primate spaces and the dental arches shape in kids from 3 to 6 years of age, from the I.E. Inicial Niño Jesús de Praga in Moyobamba, Peru, during the 2023 school year.

**Materials and methods:**

This prospective, traverse, descriptive and observational study was made up of 237 participants, who were examined using a clinical file where the findings were recorded based on, he absence or presence of primate spaces and the shape of the dental arch was classified as oval, square or triangular. A univariate analysis of frequency and percentage, and a bivariate analysis was carried out using chi square, with a significance level of 0.05.

**Results:**

It was found that in the upper arch 82.3% presented primate spaces in both quadrants, in the lower arch it was 50.2% followed by 45.6% that showed absence of primate spaces in both quadrants. The most common dental arch shape in both arches was oval (70.5%). 58.6% presented primate spaces in both quadrants and an oval arch shape for the upper arch and 34.6% for the lower arch. A statistically significant relationship was found between primate spaces and dental arch shape in the upper arch (P=0.005), of 4-year-old (P=0.025) and female participants (P=0.002).

**Conclusion:**

The presence of primate spaces in both quadrants was predominant for the upper and lower arch, as well as the oval arch shape, with the combination of these characteristics being the most frequent, so the development of its occlusion is expected to be harmonious.

## INTRODUCCIÓN

Las alteraciones oclusales son encontradas con frecuencia en la práctica clínica y representan un reto mayor para el tratamiento cuando los pacientes son abordados a una edad adulta. Por ello, los odontólogos deben estar en la capacidad de diagnosticar las alteraciones de la oclusión, por lo que resulta necesario comprender las diferentes etapas por las que transita la dentición ^1^. El tratamiento anticipado tiene como objetivo corregir las discrepancias esqueléticas, dentoalveolares y musculares, antes de que finalice la erupción de los dientes permanentes, con el propósito de lograr un entorno orofacial armonioso. Solo reconociendo la etiología será posible establecer y definir las metas del tratamiento ^2^. La observación de las características de la dentición decidua durante los años formativos contribuye a predecir las características de la dentición futura ^3^.

El desarrollo de los maxilares y los dientes se inicia en la vida intrauterina. El crecimiento de los maxilares es dinámico, pues al momento del nacimiento la mandíbula se encuentra retruída, lo cual va cambiando durante la lactancia ^(4, 5)^ Por su parte, los gérmenes dentarios observados oclusalmente presentan un apiñamiento, reconocido como apiñamiento embrionario primitivo, atribuido a la lámina dentaria y su patrón de crecimiento ^6^. La lactancia va estimulando el crecimiento de la región orofacial ^7^, que es ortopédico, y se vuelve el primer proceso de ortopedia natural funcional de los maxilares ^8^.

El sistema óseo que da soporte a los órganos dentales es conocido como arco dentario ^2^. En conjunto, el tejido óseo, la erupción dental, el tejido muscular orofacial y las fuerzas intraorales son los agentes que actúan en los patrones de forma adoptados por los arcos dentarios, así como también se suman los hábitos parafuncionales, las enfermedades sistémicas y el tipo de alimentos que conforman la dieta. Además, no podemos dejar de mencionar el factor genético, que determina la longitud y el ancho ^9^. En consecuencia, la forma que adoptan los arcos dentarios es el resultado de las fuerzas ejercidas durante la funcionalidad del sistema estomatognático, influenciado por la actividad muscular, la anatomía de los dientes, así como su posición y dimensión, los cuales actúan como promotores en el proceso crecimiento y remodelación ^10^. Basándonos en el concepto de que las arcadas dentales son simétricas y podrían ser representadas mediante formas geométricas, en diversos grupos humanos se identificaron múltiples formas y tamaños de arcos dentarios. Entre las más estudiadas y documentadas están la forma de arco ovoide, que presenta mayor prevalencia; la forma de arco cuadrada, cuando el ancho es en forma de U; y la forma triangular, cuando se presenta largo y angosto en forma de V ^9^^,^^10^.

Los espacios fisiológicos son características de la dentición decidua ^4^^,^^11^ y representan un mecanismo de compensación de la discrepancia mesiodistal entre los incisivos de ambas denticiones. Su ausencia permite realizar el diagnóstico oportuno de futuras anomalías; además, pueden estar relacionados con micrognatismo transversal del maxilar o macrodoncia de los dientes deciduos ^5^^,^^12^, lo que evidencia la desproporción existente entre los maxilares y el tamaño de los dientes ^13^.

Los espacios primate son los espacios fisiológicos más amplios y prevalentes ^14^. Se identificó su presencia a partir de los primates (simios) durante la dentición decidua y permanente, como proyección de los caninos ^15^; por ello, se señala que son de origen congénito y presentan un patrón inherente al momento de la erupción de los caninos deciduos, antes que una adaptación funcional evolutiva ^5^^,^^7^^,^^14^. Se encuentran ubicados entre la cara distal del incisivo lateral y la cara mesial del canino, en la arcada superior, mientras que en la arcada inferior se ubican entre la cara distal del canino y la cara mesial del primer molar ^16^ (figura 1). En cuanto a la arcada superior, los espacios primate generan una ganancia de la distancia intercanina, así como ayudan a equilibrar la diferencia de tamaño de los incisivos. Mientras que, en la arcada inferior, algunos autores indican que, al erupcionar el incisivo lateral permanente, los espacios primate permiten que el canino temporal se distalice y preserve el espacio para su sucesor. Por otro lado, se afirma que el espacio se cierra tras la erupción del primer molar permanente, el cual ejerce presión hacia mesial ^17^.

Las interacciones complejas entre los factores genéticos y ambientales cumplen un rol primordial en el comportamiento de las variables estudiadas. Mediante estudios longitudinales, se ha intentado demostrar la divergencia entre el tamaño de dientes y arcos dentarios en los últimos 40 años ^18^. Por ese motivo, se recogió información de la forma de arco dental y los espacios primate, a fin de evaluar su comportamiento según edad, sexo y arcadas, valorando la relación que presentan, así como en busca de un posible factor de predictibilidad, asociado a patrones fisiológicos propios de cada individuo y de una comunidad. El conocimiento de la interacción de ambas variables juega un papel importante en la prevención; además, el cuidado, la vigilancia y el control resultan vitales para la atención primaria de salud. Entonces, establecer una base de datos, relacionarlos e interpretar los resultados impulsa la creación de estrategias de salud públicas efectivas, basadas en las características propias de la población.

El presente estudio tuvo como propósito establecer una relación entre los espacios primate y la forma de arcos dentarios en niños de 3 a 6 años de la I. E. Inicial Niño Jesús de Praga, de la ciudad de Moyobamba, Perú.

## MATERIALES Y MÉTODOS

El presente estudio transversal fue aprobado por el Comité Metodológico Institucional de Bioética en Investigación en Seres Humanos de la Facultad de Medicina de la Universidad Católica Santo Toribio de Mogrovejo, de Chiclayo (Perú), mediante Resolución N.° 141-2023-USAT-FMED.

La población estuvo conformada por todos los estudiantes de 3 a 6 años matriculados en la I. E. Inicial Niño Jesús de Praga, de la ciudad de Moyobamba, en el año escolar 2023, con un total de 249 niños; este dato fue obtenido a partir de la dirección de la institución educativa. Los participantes seleccionados en el estudio cumplieron los siguientes criterios de inclusión: niños de 3 a 6 años que estudian en la I. E. Inicial Niño Jesús de Praga durante el periodo escolar 2023; niños cuyos padres de familia hayan firmado el consentimiento informado; niños que brinden su consentimiento para la evaluación; niños en fase de dentición decidua; niños que no presenten alteraciones de número, forma, estructura ni presenten lesiones de caries interproximales. Finalmente, participaron en el estudio 237 niños.

El investigador fue calibrado en tres sesiones por un experto (GP) en dentición decidua, con lo que se obtuvo un valor de Kappa de 0,81, calificado como valor de concordancia bueno, con un nivel de significancia estadística de p < 0,01.

La recolección de datos se realizó a través de una ficha clínica que le permitió al examinador registrar información en función a las variables estudiadas; así, para espacios primate en la arcada superior se registró su presencia en ambos cuadrantes, ausencia en ambos cuadrantes, presente solo en el cuadrante I o el cuadrante II. Asimismo, en la arcada inferior se registró su presencia en ambos cuadrantes, ausencia en ambos cuadrantes, presente solo en el cuadrante III y presente solo en el cuadrante IV. Los arcos dentarios se registraron según su forma (ovalado, triangular o cuadrado), tanto la arcada superior como la inferior. La ficha clínica también permitió el registro de la edad y el sexo de los participantes. Para realizar la evaluación clínica, el examinador programó la visita de un aula por turno al inicio de la jornada pedagógica. En cuanto el participante, se encontró en su aula y el examinador, tras verificar la autorización mediante la firma del consentimiento informado por parte del padre de familia, además de obtener el consentimiento del participante, procedió a realizar el examen clínico, para lo cual contó con el EPP correspondiente, la ayuda de un baja lengua descartable y una linterna de luz blanca.

### Plan de análisis

Los datos obtenidos en la ficha clínica fueron tabulados y procesados a través de un análisis univariado de frecuencias y porcentajes, así como el análisis bivariado mediante la prueba estadística de chi cuadrado, con un nivel de significancia de 0,05, para determinar la asociación entre variables. Los datos fueron analizados estadísticamente por medio del programa SPSS para Windows versión 19.

## RESULTADOS

En la Tabla 1 se evidencia que el estudio contó con un total de 237 participantes, en su mayoría niños de 4 y 5 años. Contó con más participantes de sexo masculino (52.3%), pero en el grupo de 5 años se contó con más participantes del sexo femenino, mientras que el número de participantes de 6 años fue igual en ambos sexos.

**Tabla 1:** Edad y sexo de los participantes

EDAD	SEXO				TOTAL	
	Masculino		Femenino			
	n	%	n	%	n	%
3	35	14,8	22	9,3	57	24,1
4	44	18,6	41	17,3	85	35,9
5	39	16,5	44	18,6	83	35
6	6	2,5	6	2,5	12	5,1
TOTAL	124	52,3	113	47,7	237	100

En la Tabla 2, la arcada superior muestra que gran parte de los participantes presentaron espacios primate en ambos cuadrantes (82,3%) y forma de arco ovalado (70,5%), siendo el 58,6% quienes presentan ambas características. La prueba de chi cuadrado encontró relación estadísticamente significativa para las variables en la arcada superior (p = 0,005). Por su parte, en la arcada inferior, el 50,2% de los participantes presentaron espacios primate en ambos cuadrantes, seguidos por el 45,6% que no presentaron espacios primate en ningún cuadrante. Asimismo, el 34,6% de los participantes mostraron presencia de espacios primate en ambos cuadrantes y forma de arco ovalado, seguidos por los que mostraron ausencia de espacios primate en ambos cuadrantes y forma de arco ovalado (32,1%). La prueba de chi-cuadrado no encontró relación estadísticamente significativa entre las variables en la arcada inferior (p = 0,426).

**Tabla 2:** Relación entre espacios primate y la forma de arcos dentarios en los niños de 3 a 6 años de la I. E. Inicial Niño Jesús de Praga, de la ciudad de Moyobamba, 2023

ARCADA	ESPACIOS PRIMATE	FORMA DE ARCO								P
		Ovalado		Triangular		Cuadrado		Total			
		n	%	n	%	n	%	n	%		
Superior	Presente en ambos cuadrantes	139	58,6	2	0,8	54	22,8	195	82,3	0,005
	Ausente en ambos cuadrantes	22	9,3	4	1,7	7	3	33	13,9	
	Presente solo en cuadrante I	2	0,8	0	0	3	1,3	5	2,11	
	Presente solo en cuadrante II	4	1,7	0	0	0	0	4	1,69	
	Total	167	70,5	6	2,5	64	27	237	100		
Inferior	Presente en ambos cuadrantes	82	34,6	1	0,4	36	15,2	119	50,2	0,426
	Ausente en ambos cuadrantes	76	32,1	5	2,1	27	11,4	108	45,6	
	Presente solo en cuadrante III	3	1,3	0	0	0	0	3	1,27	
	Presente solo en cuadrante IV	6	2,5	0	0	1	0,4	7	2,95	
	Total	167	70,5	6	2,53	64	27	237	100		

*Prueba de chi-cuadrado

La Tabla 3 muestra que, en los participantes de 3 años, predominan los espacios primate en ambos cuadrantes y forma de arco dentario ovalado en la arcada superior (68,4%), mientras que para la arcada inferior hay predominio de ausencias de espacios primate en ambos cuadrantes y forma de arco ovalado (42,1%). En cuanto a los participantes de 4 años, tanto en la arcada superior como la inferior, se encontró un predominio de espacios primate en ambos cuadrantes y forma de arco ovalado (55,3% y 36,5%). Asimismo, en los participantes de 5 años, se evidencia un predomino de espacios primate en ambos cuadrantes y forma de arco ovalado en la arcada superior (55,4%), mientras que para la arcada inferior la diferencia entre presencia y ausencia de espacios primates en ambos cuadrantes y forma de arco ovalado es de 2 participantes, lo que representa el 32,5% y el 30,1%, respectivamente. En tanto, los participantes de 6 años, así como los demás grupos etarios, para la arcada superior, mostraron una prevalencia de espacios primate en ambos cuadrantes y forma de arco ovalado (58,3%), mientras que, en la arcada inferior, el 33,3% no presentaron espacios primate en ningún cuadrante y forma de arco ovalado, así como forma de arco cuadrado. En todos los grupos etarios, la forma de arco predominante en ambas arcadas fue la ovalada, y ningún participante de 3 y 6 años presentó forma de arco triangular. La prueba de chi-cuadrado solo mostró relación estadísticamente significativa entre espacios primate y forma de arco dentario en la arcada superior de los niños de 4 años (p = 0,025).

**Tabla 3:** Relación entre espacios primate y la forma de arco, según edad de niños en la I. E. Inicial Niño Jesús de Praga

EDAD	ARCADA	ESPACIOS PRIMATE	FORMA DE ARCO								P
			Ovalado		Triangular		Cuadrado		Total		
			n	%	n	%	n	%	n	%	
3 años	Superior	Presente en ambos cuadrantes	39	68,4	0	0	10	17,5	49	86	0,822
		Ausente en ambos cuadrantes	6	10,5	0	0	1	1,8	7	12,3	
		Presente solo en cuadrante I	0	0	0	0	0	0	0	0	
		Presente solo en cuadrante II	1	1,8	0	0	0	0	1	1,8	
		Total	46	80,7	0	0	11	19,3	57	100	
	Inferior	Presente en ambos cuadrantes	21	36,8	0	0	7	12,3	28	49,1	0,529
		Ausente en ambos cuadrantes	24	42,1	0	0	4	7,0	28	49,1	
		Presente solo en cuadrante III	0	0	0	0	0	0	0	0	
		Presente solo en cuadrante IV	1	1,8	0	0	0	0	1	1,8	
		Total	46	80,7	0	0	11	19,3	57	100	
4 años	Superior	Presente en ambos cuadrantes	47	55,3	2	2,4	20	23,5	69	81,2	0,025
		Ausente en ambos cuadrantes	7	8,2	3	3,5	1	1,2	11	12,9	
		Presente solo en cuadrante I	1	1,2	0	0	2	2,4	3	3,5	
		Presente solo en cuadrante II	2	2,4	0	0	0	0	2	2,4	
		Total	57	67,1	5	5,9	23	27,1	85	100	
	Inferior	Presente en ambos cuadrantes	31	36,5	1	1,2	15	17,6	47	55,3	0,361
		Ausente en ambos cuadrantes	23	27,1	4	4,7	7	8,2	34	40	
		Presente solo en cuadrante III	0	0	0	0	0	0	0	0	
		Presente solo en cuadrante IV	3	3,5	0	0	1	1,2	4	4,7	
		Total	57	67,1	5	5,9	23	27,1	85	100	
5 años	Superior	Presente en ambos cuadrantes	46	55,4	0	0	20	24,1	66	79,5	0,446
		Ausente en ambos cuadrantes	9	10,8	1	1,2	4	4,8	14	16,9	
		Presente solo en cuadrante I	1	1,2	0	0	1	1,2	2	2,4	
		Presente solo en cuadrante II	1	1,2	0	0	0	0	1	1,2	
		Total	57	68,7	1	1,2	25	30,1	83	100	
	Inferior	Presente en ambos cuadrantes	27	32,5	0	0	13	15,7	40	48,2	0,736
		Ausente en ambos cuadrantes	25	30,1	1	1,2	12	14,5	38	45,8	
		Presente solo en cuadrante III	3	3,6	0	0	0	0	3	3,6	
		Presente solo en cuadrante IV	2	2,4	0	0	0	0	2	2,4	
		Total	57	68,7	1	1,2	25	30,1	83	100	
6 años	Superior	Presente en ambos cuadrantes	7	58,3	0	0	4	33,3	11	91,7	0,217
		Ausente en ambos cuadrantes	0	0	0	0	1	8,3	1	8,3	
		Presente solo en cuadrante I	0	0	0	0	0	0	0	0	
		Presente solo en cuadrante II	0	0	0	0	0	0	0	0	
		Total	7	58,3	0	0	5	41,7	12	100	
	Inferior	Presente en ambos cuadrantes	3	25	0	0	1	8,3	4	33,3	0,408
		Ausente en ambos cuadrantes	4	33,3	0	0	4	33,3	8	66,7	
		Presente solo en cuadrante III	0	0	0	0	0	0	0	0	
		Presente solo en cuadrante IV	0	0	0	0	0	0	0	0	
		Total	7	58,3	0	0	5	41,7	12	100	

*Prueba de chi-cuadrado

Por su parte, la Tabla 4 muestra que un mayor número de participantes de sexo masculino cuentan con espacios primate en ambos cuadrantes, tanto en la arcada superior como en la inferior (83,9% y 57,3%), de los cuales el 60,5% y el 37,9% presentan forma de arco ovalado. Mientras que, en el sexo femenino, en la arcada inferior, el 51,3% de las participantes no presentaron espacios primate en ningún cuadrante, y de estas el 31,9% presentaron forma de arco ovalado y solo el 2,7%, forma de arco triangular. Tanto para el sexo masculino como el femenino, la forma de arco dentario predominante en la arcada superior e inferior fue de la forma ovalada, seguida por la cuadrada. La prueba de chi-cuadrado solo mostró relación estadísticamente significativa entre espacios primate y forma de arco dentario en la arcada superior del sexo femenino (p = 0,002).

**Tabla 4:** Relación entre espacios primate y la forma de arco, según sexo en niños de 3 a 6 años de la I. E. Inicial Niño Jesús de Praga

SEXO	ARCADA	ESPACIOS PRIMATE	FORMA DE ARCO								P
			Ovalado		Triangular		Cuadrado		Total			
			n	%	n	%	n	%	n	%		
Masculino	Superior	Presente en ambos cuadrantes	75	60,5	2	1,6	27	21,8	104	83,9	0,716
		Ausente en ambos cuadrantes	10	8,1	1	0,8	3	2,4	14	11,3	
		Presente solo en cuadrante I	1	0,8	0	0	1	0,8	2	1,6	
		Presente solo en cuadrante II	4	3,2	0	0	0	0	4	3,2	
		Total	90	72,6	3	2,4	31	25	124	100	
	Inferior	Presente en ambos cuadrantes	47	37,9	1	0,8	23	18,5	71	57,3	0,460
		Ausente en ambos cuadrantes	40	32,3	2	1,6	8	6,5	50	40,3	
		Presente solo en cuadrante III	1	0,8	0	0	0	0	1	0,8	
		Presente solo en cuadrante IV	1	0,8	0	0	1	0,8	2	1,6	
		Total	89	71,8	3	2,4	32	25,8	124	100	
Femenino	Superior	Presente en ambos cuadrantes	64	56,6	0	0	27	23,9	91	80,5	0,002
		Ausente en ambos cuadrantes	12	10,6	3	2,7	4	3,5	19	16,8	
		Presente solo en cuadrante I	1	0,9	0	0	2	1,8	3	2,7	
		Presente solo en cuadrante II	0	0	0	0	0	0	0	0	
		Total	77	68,1	3	2,7	33	29,2	113	100	
	Inferior	Presente en ambos cuadrantes	35	31	0	0	13	11,5	48	42,5	0,345
		Ausente en ambos cuadrantes	36	31,9	3	2,7	19	16,8	58	51,3	
		Presente solo en cuadrante III	2	1,8	0	0	0	0	2	1,8	
		Presente solo en cuadrante IV	5	4,4	0	0	0	0	5	4,4	
		Total	78	69	3	2,7	32	28,3	113	100	

*Prueba de chi-cuadrado

La Tabla 5 muestra que el 48,5% presentaron espacios primate en ambos cuadrantes tanto en la arcada superior como en la inferior, seguido de la presencia de espacios primate en ambos cuadrantes en la arcada superior, con ausencia de espacios primate en ambos cuadrantes en la arcada inferior (30,8%). La prueba de chi-cuadrado muestra una relación estadísticamente significativa entre los espacios primate de la arcada superior y la arcada inferior (p = 0,001). En cuanto a la forma de arco, predominó la formar ovalada (70%), seguida por la forma cuadrada (26,6%). La forma de arco de la arcada superior e inferior mostró una relación estadísticamente significativa mediante la prueba de chi-cuadrado (p = 0,001).

**Tabla 5:** Relación entre espacios primate y la forma de arco, según arcada superior e inferior en niños de 3 a 6 años de la I. E. Inicial Niño Jesús de Praga

RELACIÓN ENTRE ARCADAS					
VARIABLES	ARCADA SUPERIOR	ARCADA INFERIOR	F	%	P
ESPACIO PRIMATE	Presente en ambos cuadrantes	Presente en ambos cuadrantes	115	48,5	0,001
		Ausente en ambos cuadrantes	73	30,8	
		Presente solo en cuadrante III	1	0,4	
		Presente solo en cuadrante IV	6	2,5	
	Ausente en ambos cuadrantes	Presente en ambos cuadrantes	2	0,8	
		Ausente en ambos cuadrantes	29	12,2	
		Presente solo en cuadrante III	2	0,8	
		Presente solo en cuadrante IV	0	0	
	Presente solo en cuadrante I	Presente en ambos cuadrantes	1	0,4	
		Ausente en ambos cuadrantes	3	1,3	
		Presente solo en cuadrante III	0	0	
		Presente solo en cuadrante IV	1	0,4	
	Presente solo en cuadrante II	Presente en ambos cuadrantes	1	0,4	
		Ausente en ambos cuadrantes	3	1,3	
		Presente solo en cuadrante III	0	0	
		Presente solo en cuadrante IV	0	0	
FORMA DE ARCO	Ovalado	Ovalado	166	70	0,001
		Triangular	0	0	
		Cuadrado	1	0,4	
	Triangular	Ovalado	0	0	
		Triangular	6	2,5	
		Cuadrado	0	0	
	Cuadrado	Ovalado	1	0,4	
		Triangular	0	0	
		Cuadrado	63	26,6	

*Prueba de chi-cuadrado

## DISCUSIÓN

El propósito del presente estudio fue determinar la relación entre los espacios primate y la forma de los arcos dentarios en niños de 3 a 6 años de la I. E. Inicial Niño Jesús de Praga, de la ciudad de Moyobamba, para lo cual se contó con la participación de 237 alumnos, de los cuales el 52,3% fue de sexo masculino; asimismo, el 24,1% tenía 3 años; el 35,9%, 4 años; el 35%, 5 años, y el 5,1%, 6 años.

El estudio mostró mayor presencia de espacios primate en la arcada superior, con el 82,3%, mientras que en la arcada inferior solo apareció en el 50,2%, cifra que se acerca a la reportada por Meléndez *et al*. ^19^, quienes, en un estudio realizado en la ciudad de Coahuila (México), evaluaron los modelos de estudio de niños de 3 a 5 años y hallaron una prevalencia de espacios primate en la arcada superior (73,3%) frente a la arcada inferior (60%). Del mismo modo, Sun *et al*. ^16^, en un estudio realizado en niños de 3 a 6 años de Taiwán, reportan la prevalencia de espacios primate en la arcada superior (83,7%) ante la arcada inferior (61,2%). En ambos estudios se destaca la prevalencia de espacios primates en la arcada superior frente a la inferior. Al igual que en el presente estudio, dichos autores coinciden que la presencia de espacios primate es más frecuente en el sexo masculino. 

En concordancia con los resultados que se obtuvieron en el estudio, Lochib *et al*. ^20^, teniendo en cuenta las características oclusales y la prevalencia de anomalías dentales, hallaron que el 61,7% de los participantes presentaron espacios primate en la arcada superior y el 27,9%, en la arcada inferior. Por su parte, Bahadure *et al*. ^21^, quienes estudiaron los rasgos oclusales de la dentición temporal de niños en edad preescolar en la India, reportaron que el 64,7% de los participantes presentó espacios primate en la arcada superior y el 50,7%, en la arcada inferior, y fue estadísticamente significativo en los niños de 5 años. El presente estudio encontró relación estadísticamente significativa entre los espacios primate de la arcada superior e inferior, pero solo halló una relación estadísticamente significativa entre espacios primate y forma de arco dentario en la arcada superior de los niños de 4 años, y no en los demás grupos etarios evaluados. 

En cuanto a la presencia de espacios primate en los cuadrantes, el estudio mostró que, tanto en la arcada superior como en la inferior, hubo mayor presencia de espacios primate en ambos cuadrantes a la vez, siendo estos los cuadrantes I y II para la arcada superior, y III y IV para la arcada inferior, seguidos por la ausencia de espacios primate en ambos cuadrantes, lo que difiere de lo encontrado por Rokan ^3^, quien, en un estudio realizado en Iraq, evaluó a 70 niños de 4 a 5 años, y reportó que, en la arcada inferior, el 77,14% presentaron espacios primate solo en la hemiarcada izquierda, en comparación con el presente estudio, en el cual solo el 1,3% presentan espacios primate únicamente en el cuadrante III.

Srinivasan *et al*. ^22^ evaluaron espacios primate en niños de 3 a 5 años de la India, y concluyeron que estos espacios son significativos en los tres grupos de edad, tanto en los arcos superiores como los inferiores. Por otra parte, el presente estudio identificó que, en la arcada inferior, el mismo número de participantes de 3 años mostraron ausencia y presencia de espacios primate, y en el caso de los de 6 años el número de participantes que no presentan espacios primate es mayor al que sí lo presenta. Solo se encontró relación estadísticamente significativa entre espacios primate y forma de arco dentario en la arcada superior en los participantes de 4 años. 

Esto contrasta con un estudio realizado en Chachapoyas (Perú) por Flores ^15^, en el cual evaluó la relación entre espacios primate y apiñamiento dental, y encontró que el 17,1% presentaron espacios primates en ambas arcadas, lo que en el presente estudio representó el 48,5%. Asimismo, el 6,7 % lo presentó solo en la arcada superior y el 2,9% en la arcada inferior, mientras que en el presente estudio esos porcentajes fueron del 30,8% y el 0,8%, respectivamente. Por otro lado, en la población de Chachapoyas, el 25,7% no presentó espacios primate, en comparación con el estudio, en el cual el 12,2% mostró ausencia de espacios primate. Es preciso considerar que la población estudiada por Flores tuvo entre 3 y 8 años; además, no encontró una relación estadísticamente significativa entre espacios primate y sexo de los participantes. Si bien el presente estudio buscó una relación entre espacios primate y forma de arco, la relación estadística fue significativa en la arcada superior de las participantes de sexo femenino. 

Con respecto a la forma de arco, el estudio en mención mostró una prevalencia de forma de arco ovalado (70,5%), lo que coincide con Zúñiga e Ibárcena ^7^, quienes evaluaron a 58 niños de entre 3 y 6 años de la ciudad de Puno, Perú, y encontraron que el 81% presentó forma de arco ovalado. Por su parte, Mendoza *et al*. ^23^, en un estudio llevado a cabo en México, identificaron que la forma de arco más frecuente, tanto para el sexo femenino como el masculino, fue el ovalado en la arcada superior, mientras que en la arcada inferior fue el triangular, lo cual discrepa de lo encontrado en el presente estudio, ya que ambos sexos en ambas arcadas presentaron forma de arco ovalado, seguida por la cuadrada y, en muy pocos casos, la triangular. 

Asimismo, al revisar el estudio realizado por Orozco *et al*. ^9^, quienes evaluaron modelos de estudio de pacientes de la clínica multidisciplinaria de Zaragoza, se encuentra que el 64% presentan forma de arco cuadrada en la arcada superior, mientras que el 46,7% de los arcos fueron ovalados en la arcada inferior, seguido por el 42% que fueron cuadrados, y los mismos patrones se repiten en ambos sexos. El estudio concluye que la forma de arco predominante para este grupo étnico fue la cuadrada, lo cual está en discordancia con lo reportado en el presente estudio, donde, en ambas arcadas y sexos, el 70,5% presentan forma de arco ovalado; el 27%, forma de arco cuadrado, y el 2,5%, forma triangular. Por el contrario, sí encuentra semejanza con Rivera *et al*. ^24^, quienes evaluaron a niños de una comunidad indígena en Leticia, Colombia, y encontraron que en la arcada superior el 86% presentó forma de arco ovalado y el 14%, cuadrado; mientras que en la arcada inferior el 75% presentó forma de arco ovalado y el 25%, cuadrado. No encontraron datos para la forma de arco triangular, y se repitió la frecuencia para ambos sexos, por lo cual concluyen que la forma de arco ovalado es predominante en este grupo étnico. Los resultados obtenidos en estos estudios refuerzan el concepto de que las formas de los arcos dentales están influenciadas por diferentes componentes genéticos y ambientales, desde factores de crecimiento, genéticos, hábitos alimenticios, parafuncionales e influencia y costumbres propias de la comunidad. 

Para recapitular, en el presente estudio, el 70,5% presentó forma de arco ovalado en ambas arcadas; el 82,3%, presencia de espacios primate en ambos cuadrantes en la arcada superior, y el 50,2%, en la arcada inferior. Fue más prevalente en el sexo masculino, que constituyó el 52,3% de la muestra, y en los participantes de 4 y 5 años, quienes representan el 35,9% y 35%, respectivamente. Esto concuerda con Chamba ^11^, quien, en un estudio realizado en Loja, Ecuador, indica que, de 40 niños de 5 años, el 97,5% presentó forma de arco ovoide; el 75,5%, espacios primate en la arcada superior, y se halló prevalencia en el sexo masculino. Sin embargo, no coincide completamente con lo documentado por Vásquez ^14^ en la ciudad de Sucre, Bolivia, donde el 93,85% de participantes presentaron forma de arco ovalado; el 66,15%, espacios primate en la arcada superior y en la arcada inferior, y el 84,62%, ausencia de espacio primate en la hemiarcada izquierda, característica que en el presente estudio solo presentó el 3% de la muestra. 

Finalmente, para resaltar los resultados obtenidos, un número mayor de participantes presentaron espacios primate en ambos cuadrantes y forma de arco ovalado en la arcada superior (58,6%), lo que mostró una relación estadísticamente significativa entre estas variables, mientras que, en la arcada inferior, la presencia de espacios primate en ambos cuadrantes y forma de arco ovalado fue del 34,6%, seguido por la ausencia de espacios primate en ambos cuadrantes y forma de arco ovalado, en un 32,1%, por lo que no se encontró relación estadísticamente significativa. En este punto, podemos señalar lo manifestado por Trigo y Mercado ^25^, quienes afirman que los espacios primate están más presentes en la arcada superior que en la inferior, lo cual es una característica más prevalente en el sexo masculino. Por ello, sugieren que el patrón de crecimiento facial tiene participación en el comportamiento de los espacios primate; asimismo, señalan que la dirección del crecimiento de los maxilares se ve impactado por el patrón facial. 

Debido al tamaño de la muestra, los resultados obtenidos en el presente estudio no son extrapolables a la población en general, lo que representa una limitación, por lo cual se sugiere su réplica en otras muestras que permitan contrastar estos hallazgos.

## CONCLUSIONES

La presencia de espacios primate en ambos cuadrantes fue predominante para la arcada superior e inferior, así como la forma de arco ovalado, siendo la combinación de estas características la más frecuente, por lo que se espera que el desarrollo de su oclusión sea armonioso.

## Fuente de financiamiento:

Autofinanciado
